# Distinct IDH1/2-associated Methylation Profile and Enrichment of *TP53* and *TERT* Mutations Distinguish Dedifferentiated Chondrosarcoma from Conventional Chondrosarcoma

**DOI:** 10.1158/2767-9764.CRC-22-0397

**Published:** 2023-03-14

**Authors:** Josephine Kam Tai Dermawan, Khedoujia Nafa, Abhinita Mohanty, Yingjuan Xu, Ivelise Rijo, Jacklyn Casanova, Liliana Villafania, Jamal Benhamida, Ciara M. Kelly, William D. Tap, Patrick J. Boland, Nicola Fabbri, John H. Healey, Marc Ladanyi, Chao Lu, Meera Hameed

**Affiliations:** 1 Department of Pathology and Laboratory Medicine, Memorial Sloan Kettering Cancer Center, New York, New York.; 2 Department of Medicine, Memorial Sloan Kettering Cancer Center, New York, New York.; 3 Department of Surgery, Memorial Sloan Kettering Cancer Center, New York, New York.; 4 Department of Orthopedic Surgery, New York University Grossman School of Medicine, New York, New York.; 5 Department of Genetics and Development, Columbia University Medical Center, New York, New York.

## Abstract

Dedifferentiated chondrosarcoma (DDCS) is a rare high-grade chondrosarcoma characterized by a well-differentiated chondrosarcoma (WDCS) component that abruptly transitions to a high-grade, noncartilaginous sarcomatous component. To date, the molecular pathogenesis of DDCS and its distinction from conventional chondrosarcoma remain poorly understood. By targeted sequencing, we examined the mutational and copy-number profiles of 18 DDCS, including macrodissected WDCS components, compared with 55 clinically sequenced conventional chondrosarcomas. In conjunction with publicly available external data, we analyzed the methylation and expression profiles of 34 DDCS and 94 conventional chondrosarcomas. Isocitrate dehydrogenase 1/isocitrate dehydrogenase 2 (*IDH1/IDH2)* mutations were present in 36% conventional chondrosarcomas and 71% DDCS. Compared with conventional chondrosarcomas, DDCS had higher frequencies of *TP53* and *TERT* promoter mutations and *CDKN2A/B* copy-number losses. Paired analysis of macrodissected WDCS and the high-grade components revealed *TERT* promoter mutations as early events. Despite phenotypic similarities, the percentage of genome with copy-number alterations in DDCS was significantly lower than that in other high-grade sarcomas. Differential methylation analysis revealed reduction of *IDH1/IDH2*-associated global hypermethylation characteristically seen in conventional chondrosarcoma and a distinct methylation profile in DDCS. The WDCS and high-grade components in DDCS showed similar methylation profiles. These CpG sites were associated with upregulated expression of genes involved in G_2_–M checkpoints and E2F targets. Genomic profiling revealed enrichment of *TP53*, *TERT* promoter, and *CDKN2A/B* alterations in DDCS. Integrated methylation and gene expression analysis revealed distinct *IDH1/IDH2*-associated methylation and transcriptional profiles as early events in DDCS, which may underlie the pathogenesis of dedifferentiation in chondrosarcomas.

**Significance:**

DDCS is a rare, high-grade chondrosarcoma with a dismal prognosis. About 50%–80% of DDCS harbor IDH1/IDH2 mutations. We uncover a significant alteration of IDH-associated methylation profile in DDCS, which we propose is key to the progression to dedifferentiation. In this context, the potential effect of the use of IDH inhibitors is unclear but important to address, as clinical trials of selective IDH1 inhibitors showed worse outcome in DDCS.

## Introduction

Dedifferentiated chondrosarcoma (DDCS) is a high-grade chondrosarcoma (CS) characterized histologically by a well-differentiated CS (WDCS) component that abruptly transitions to a high-grade, dedifferentiated, noncartilaginous sarcomatous component (1). Patients with DDCS carry dismal prognosis, with a 5-year disease-specific survival (DSS) of 7%–24%, which is significantly worse than grade 3 CS (5-year DSS: 42%; refs. 2–5).

In conventional CS, about 50% of cases harbor somatic mutations in isocitrate dehydrogenase 1 (*IDH1*) and isocitrate dehydrogenase 2 (*IDH2*) mutations, the majority being *IDH1* R132C/H and a smaller subset *IDH2* R172 mutations (6), which are the same mutations seen in about 80% of patients with Ollier disease and Maffucci syndrome (6–8). The presence of *IDH1/IDH2* mutations helps distinguish CS from chondroblastic osteosarcoma and DDCS from undifferentiated pleomorphic sarcoma (UPS) of bone (9, 10).

Early molecular genetic studies have demonstrated that in the same tumor, both WDCS and the high-grade, noncartilaginous sarcomatous components of DDCS share common genetic alterations in *TP53* and copy-number alterations (CNA) at focal chromosomal loci, with the high-grade component displaying additional genomic aberrations such as aneuploidy and more extended CNAs, implying the two components are derived from a single precursor (11). The same group of investigators demonstrated that 50% and 85% of DDCS harbor alterations in *CDKN2A* and *TP53*, respectively, which are nonspecific oncogenic alterations seen in many sarcoma types (12).

Subsequent studies focusing on dissecting the molecular distinction between the WDCS and high-grade sarcomatous components of DDCS cases have shown variable results. One study showed that the two components shared *TP53* and *PTEN* alterations in an isolated case of DDCS (13). A recent study of paired WDCS and high-grade components of 11 DDCS cases showed that both components harbor *IDH1/2*, *COL2A1,* and *TERT* mutations, whereas *TP53* and large-scale CNAs were more common in the high-grade component (14). This was concordant with an early study showing *TP53* mutation and LOH in *TP53* and *RB1* (15). About 50%–80% of DDCS also harbors *IDH1/IDH2* mutations, with no clear prognostic impact (12, 16, 17). A recent phase I clinical study of ivosidenib (AG-120), a selective mutant IDH1 inhibitor, in patients with advanced *IDH1*-mutant CS showed worse outcomes in DDCS compared with conventional CS, suggesting a different biology between these two histotypes despite sharing *IDH1* mutations (18).

To date, the molecular pathogenesis of DDCS and its distinction from conventional CS remain poorly understood. Using MSK-IMPACT, we examined the mutational and copy-number profiles of 17 DDCS cases, including macrodissected WDCS components when available (eight cases), in comparison with 55 conventional CS cases. As methylation is a marker of cell lineage, and conventional CS and WDCS have similar histologic features, we wanted to assess whether they share similar or distinct cell lineage or undergo alterations predisposing to a dedifferentiated phenotype through methylation analysis. To this end, we analyzed the methylation patterns in conventional, WDCS, and DDCS. Using an in-house Illumina EPIC array platform for DDCS, in conjunction with external publicly available methylation and gene expression data, we analyzed the methylation profiles of overall 33 DDCS and 94 conventional CS.

## Materials and Methods

### Case Selection and Study Cohort

Cases were identified from the Memorial Sloan Kettering Cancer Center (MSKCC) surgical pathology archives from 2013 to 2021. Written informed consent was obtained from patients for use of genomics data for research. This study was conducted in accordance with the Declaration of Helsinki and was approved by the Institutional Review Board. Criteria included cases with an explicit diagnosis of DDCS and conventional grade 1–3 CS. Clinical charts and pathology reports were reviewed to document patient age, sex, tumor site, tumor grade, and outcome data. For DDCS cases, the hematoxylin and eosin sections were manually reviewed to select and circle areas of the dedifferentiated and WDCS components, which were then macrodissected for downstream DNA sequencing and methylation profiling. Because most blocks containing well-differentiated cartilaginous components were subjected to acid-based decalcification, only in seven cases of DDCS cases were the WDCS components available for further downstream processing, with an additional case of WDCS without the corresponding high-grade dedifferentiated component.

### Survival Analysis

Survival analysis by comparison of HRs using log-rank *P* testing and visualization of Kaplan–Meier curves were performed using R packages “survminer” version 0.4.9 and “survival” version 3.2.13. Clinical charts were manually reviewed to document date of initial presentation, disease progression, and survival status. Median time (in years) to disease progression was defined as the time interval between initial presentation (presence of tumor seen radiographically or on physical examination) and the first instance of tumor recurrence or distant metastases after initial surgical resection and/or chemoradiation therapy with radiographically negative evidence of residual tumor.

### Targeted DNA Sequencing, Copy-number and Mutational Profiling and Data Analysis

Detailed descriptions of MSK-IMPACT workflow and data analysis, a hybridization capture-based targeted matched tumor-normal DNA NGS assay targeting 341 to 505 genes for solid tumor were described previously (19, 20). After excluding metastatic tumors, a total of 18 DDCS cases were sequenced (17 dedifferentiated components, of which seven also had macrodissected nondecalcified WDCS component sequenced concurrently, and one DDCS case with only WDCS component without the corresponding dedifferentiated component) and analyzed by the MSK-IMPACT pipeline. In all cases, the tumor is macrodissected and we were able to obtain a relatively pure population, estimated at 50%–70% based on pathologist assessment and variant allele frequency (VAF). Between WDCS and DDCS, the DDCS component has a higher tumor cellularity and content. In addition, clinical MSK-IMPACT data were available for 55 conventional CS.

We use the reference standard sample that is a mixed positive control pool consisting of known positive samples with different classes of alterations previously validated by MSK-IMPACT as our positive quality control sample. For every run, this reference sample was run to verify that we are able to detect hotspot gene mutations, including insertions/deletions and single-nucleotide variants at an expected VAF of 5%–20%, copy-number variants, and structural variants. For negative control, DNA from 10 normal diploid blood samples were pooled in equimolar ratios to create a mixed negative control sample, verified in previous runs to be free of tumor contamination and germline copy-number variants in target genes. We also use a PCR no template control (Qubit measurement < 1.0 ng/μL; ref. 19).

For somatic mutation calling, MSK-IMPACT uses genomic DNA from tumor samples with matched patient-derived normal samples from peripheral blood. When there is no matched normal sample or the coverage of matched normal is below 50X, tumor samples will be compared against a standard, in-batch pooled normal control derived from 10 normal formalin-fixed paraffin-embedded (FFPE) samples for variant calling (19).

For genome-wide copy-number profiles, copy-number segmentation files from 54 conventional CS, 13 DDCS, 288 leiomyosarcoma (LMS), 319 UPS, and 236 osteosarcoma were downloaded from MSK cBioPortal (21). A set of normal FFPE samples were used for reference diploid genome comparison. Normalized coverage values from tumor samples were divided by corresponding values in normal samples, and log-transformed to yield log-ratios. The criteria for gene amplification and deletions are as follows: if the fold change is greater than 2, it is reported as amplification. If the fold change is −2 or below, it is reported as a deletion. The IMPACT assay targets a total of 6,729 exons across 505 genes. In addition, the panel contains probes that tile the positions of 1,042 common SNPs, which mimic a low-density SNP tiling array with locations evenly distributed across the genome-coverage values. These positions are used to supplement the copy-number analysis in genomic regions where few targeted genes are located (19). Overlapping segments were derived using the “CNTools” package version 1.52.0. CNA was considered present if the absolute segmentation mean is greater or equal to 0.5. Fraction of CNA across the genome was calculated by dividing the sum of all segments with CNA by the sum of all segments across the genome.

Mutations and gene-level CNAs were visualized and summarized using the R package “ComplexHeatmap” version 2.8.0 (22).

### DNA Methylation Profiling

Details on methylation profiling were published previously (23). Briefly, genomic DNA was extracted from FFPE tissue sections. Next, 250 ng of genomic DNA was subjected to bisulfite conversion and processed on the Illumina methylation EPIC/850k platform according to manufacturer's instructions. For methylation analysis, we included a total of 18 in-house DDCS cases, of which seven samples were macrodissected WDCS components and 11 were macrodissected dedifferentiated components, using leftover DNA following MSK-IMPACT sequencing. We also recorded the *IDH1* R132 and *IDH2* R172 mutational status of these cases (14 *IDH1/IDH2-*mutated, three wildtype, one unknown).

In addition, we downloaded the following external, publicly available methylation data generated by the Illumina 450k or EPIC/850k platforms: (i) 89 cases (73 conventional CS, 16 DDCS) from an integrated molecular characterization of CS study by Nicolle and colleagues: (24) ArrayExpress # E-MTAB-7263, and (ii) 21 conventional CS cases from the Heidelberg Sarcoma Classifier study by Koelsche and colleagues: (25) Gene Expression Omnibus (GEO) # GSE140668. We also retrieved the *IDH1/IDH2* mutational status of the cases when available. Altogether, we analyzed the methylation profiles of 34 DDCS cases (18 IDH-mutant, 10 IDH-wildtype, six IDH status unknown) and 94 conventional CS cases (49 IDH-mutant, 33 IDH-wildtype, 12 IDH status unknown).

IDAT processing and data analysis on all 128 samples was performed using R version 4.1.0 and the “minfi” package version 1.38.0.24 (26). Normalization was performed using the preprocess Illumina function and probes with a detection *P* value > 0.01 were filtered, as were SNP-related probes, and probes on sex chromosomes. After probe filtering and intersecting internal and external datasets, 256,431 CpG probes remained for downstream analysis. Methylation levels were measured using beta values (ratio of the methylated probe intensity to the overall intensity—sum of methylated and unmethylated probe intensities) for all cases. CpG probes were annotated using the “IlluminaHumanMethylationEPICanno.ilm10b4.hg19” package version 0.6.0.

### Gene Expression Profiling

External, publicly available mRNA expression profiling data of 89 CS cases [73 conventional CS (35 IDH-mutant, 26 IDH-wildtype), 16 DDCS (9 IDH-mutant, 5 IDH-wildtype)] generated by the Affymetrix Human Gene 2.0 ST Array platform from the integrated molecular characterization of CS study by Nicolle and colleagues were downloaded from ArrayExpress # E-MTAB-7264 (24).

Gene expression microarray data were imported using R version 4.1.0 and the “ArrayExpress” package version 1.56.0. Normalization and background correction were performed using the preprocess Robust Multichip Average (RMA) algorithm from the “oligo” package version 1.60.0. Probes were annotated using the Affymetrix hugene20 annotation data from the “hugene20sttranscriptcluster.db” package version 8.8.0.

**FIGURE 1:**
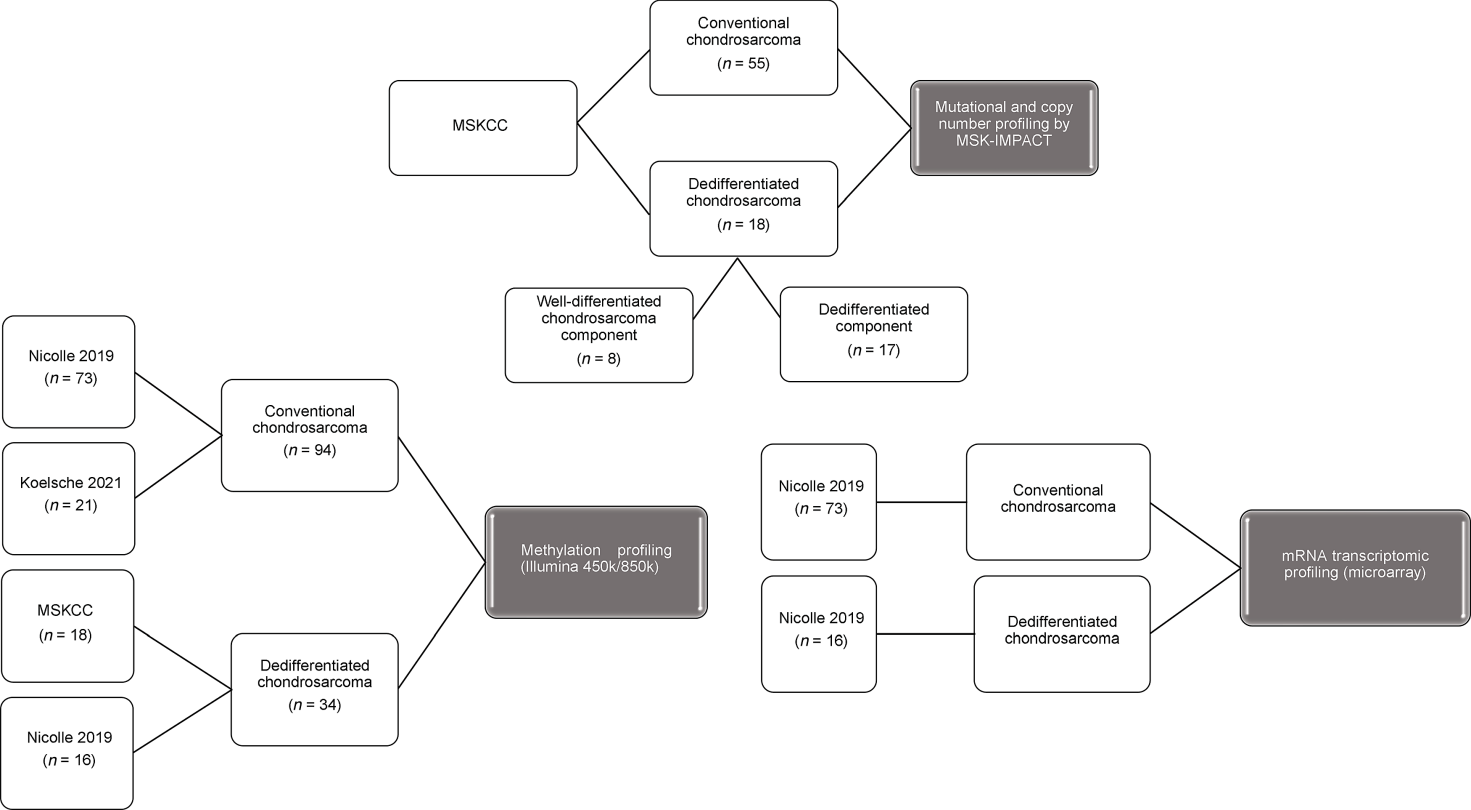
Schema outlining samples and respective sources for each analytic workflow.

A flowchart outlining the samples and respective sources for each analytic workflow is show in Fig. 1.

### Integrated Methylation and Expression Differential and Gene Set Enrichment Analysis

Differential methylation and differential gene expression analysis, respectively, between DDCS and conventional CS adjusted by IDH mutation status were performed using a two-factor (DDCS vs. conventional CS and IDH-mutant vs. IDH-wildtype) contrast matrix design with the “limma” package version 3.52.1 (27). Differentially methylated or expressed genes were computed using the empirical Bayes statistics and selected with the following parameters: *P*-value cutoff 0.05, adjusted by the Benjamini–Hochberg correction method. We defined hypomethylated and hypermethylated genes based on the t-statistic from the output of the “decideTests” function: a t-statistic of −1, 0, or 1 is classified as significantly negative, not significant, or significantly positive, respectively. The same applies for differential gene expression analysis. Thereafter, genes corresponding to differentially methylated probes were matched to differentially expressed genes by intersecting hypermethylated genes to downregulated genes (decreased expression), and hypomethylated to upregulated genes (increased expression).

Gene set enrichment analysis (GSEA) was performed using gene sets downloaded from the Molecular Signatures Database (28, 29). Gene ontology analysis and gene sets testing for methylation data were conducted using the “missMethyl” package version 1.30.0. Gene sets testing and visualization of mRNA expression data were performed using the “clusterProfiler” package version 4.4.4 using default parameters (30).

Unsupervised hierarchical clustering and heatmap generation were performed using the “ComplexHeatmap” R package version 2.12.0 with Euclidean distance for clustering of rows and columns (21). All data analysis was performed using R version 4.1.0.

### Data Availability

The data generated in this study are publicly available in GEO GSE214180.

## Results

### Clinicopathologic Summary

Among in-house cases from the MSKCC archives, 55 conventional CS and 18 DDCS cases (total 73 patients) were included in the cohort for MSK-IMPACT profiling. Patients with DDCS had a median age of 64 years old (range, 35–76) and were significantly older than those with conventional CS, who had a median age of 52 years old (range, 17–79; Student *t* test, *P* = 0.006). DDCS tended to arise from proximal appendicular skeleton (50%), for example, femur, humerus, followed by the axial skeleton (39%). In contrast, conventional CS most commonly arose from the axial skeleton (60%) followed by proximal appendicular skeleton and the head and neck (mostly skull base and larynx) at equal proportions (both at 18%). Detailed breakdown of patient characteristics is shown in Table 1.

**TABLE 1:** Patient characteristics (MSKCC cohort)

	**Conventional chondrosarcoma**		**Dedifferentiated chondrosarcoma**		**All chondrosarcoma**	
	**Count**	**% Total**	**Count**	**% Total**	**Count**	**% Total**
**Age group** ^a^						
Older than 40 years old	36	65.5%	17	94.4%	53	72.6%
Younger than 40 years old	19	34.5%	1	5.6%	20	27.4%
**Sex**						
Female	20	36.4%	10	55.6%	30	41.1%
Male	35	63.6%	8	44.4%	43	58.9%
**Primary site**						
Appendicular, distal	2	3.6%	1	5.6%	3	4.1%
Appendicular, proximal	10	18.2%	9	50.0%	19	26.0%
Axial	33	60.0%	7	38.9%	40	54.8%
Head and neck	10	18.2%	1	5.6%	11	15.1%
**Tumor grade**						
I	17	30.9%			17	23.6%
II	36	65.5%			36	50.0%
III	2	3.6%			2	2.8%
**Total**	**55**	**75.3%**	**18**	**24.7%**	**73**	**100.0%**

^a^Median age: 1. conventional CS: 52 (17–79), 2. DDCS: 64 (35–76), 3. all CS: 57.5 (17–79) years old.

### Mutational and Copy-number Profiling of DDCS versus Conventional CS

Using targeted DNA sequencing by MSK-IMPACT, we examined the mutational and copy-number profiles of 17 of 18 DDCS cases with available macrodissected dedifferentiated components, in comparison with 55 conventional CS cases. *IDH1*/*IDH2* mutations were more enriched in DDCS: present in 71% (12/17: eight *IDH1-* and four *IDH2-*mutant cases) of DDCS cases and in 36% (20/55: 17 *IDH1-* and three *IDH2-*mutant cases) of conventional CS (two-tailed *χ*^2^*P* = 0.02). In addition, compared with conventional CS, DDCS also harbored higher frequencies of *TP53* mutations (65% vs. 13%), *TERT* promoter mutations (41% vs. 5%), and *CDKN2A*/*CDKN2B* copy-number losses (41% vs. 7%). Interestingly, *TP53* mutations were mutually exclusive with *IDH1*/*IDH2* mutation only in conventional CS but not in DDCS. Overall, DDCS had higher tumor mutation burden compared with conventional CS.

**FIGURE 2:**
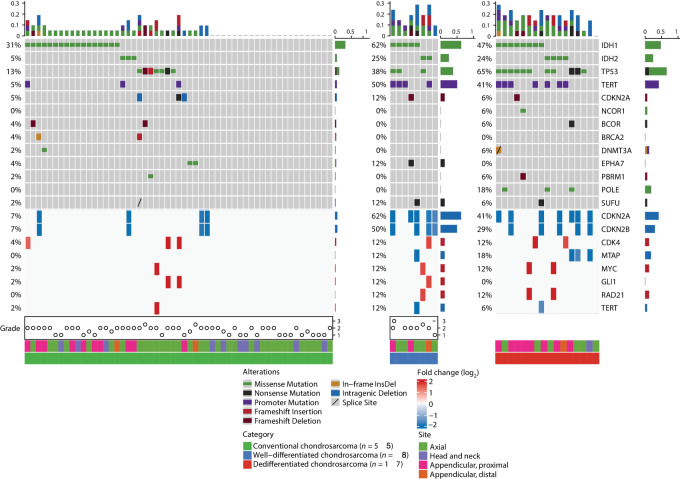
Mutational and copy-number profiling of conventional CS and DDCS. A total of 55 conventional CS, eight macrodissected WDCS components of DDCS, and 17 macrodissected high-grade noncartilaginous sarcomatous components of DDCS cases were sequenced and analyzed by the MSK-IMPACT pipeline. Shown is an Oncoprint depicting the primary tumor sites, and the types and frequencies of recurrent mutations and gene-level CNAs in each of the three CS categories.

Of the 18 DDCS cases, seven had available WDCS and high-grade components for MSK-IMPACT profiling, one had only WDCS but not the high-grade component. The WDCS (*n* = 8) components showed mutational and CNAs more similar to DDCS than conventional CS (Fig. 2). Among DDCS cases, those who harbor *TERT* promoter mutations showed borderline worse overall survival (OS) but not progression-free survival (PFS) compared with those without *TERT* promoter mutations (log-rank *P* = 0.089; Supplementary Fig. S1). Other genetic alterations were not prognostic of worse PFS or OS in DDCS, most likely due to the already dismal prognosis of this tumor type.

**FIGURE 3:**
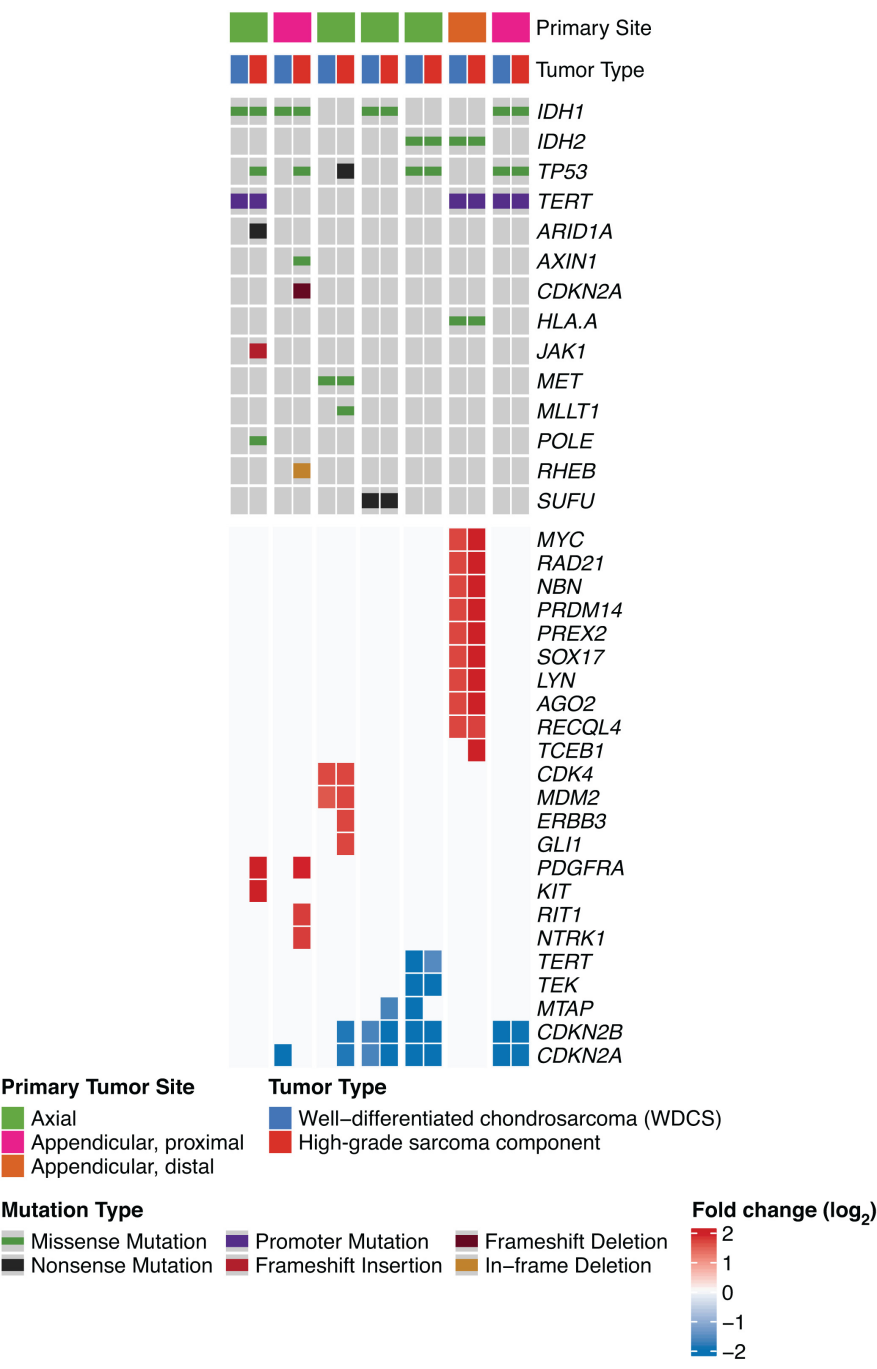
Matched mutational and copy-number profiling of cartilaginous and sarcomatous components of DDCS. Paired analysis of matched, microdissected, WDCS, and high-grade noncartilaginous sarcomatous components of 7 patients with DDCS were sequenced and analyzed by the MSK-IMPACT pipeline. Shown is an Oncoprint depicting the types and frequencies of recurrent mutations and gene-level CNAs in both components of the seven cases.

Paired analysis of macrodissected WDCS and the high-grade sarcoma components from 7 patients with DDCS revealed *TERT* promoter mutations as common, early events. On the other hand, in 3 patients, *TP53* mutations were detected only in the dedifferentiated but not the WDCS components. Furthermore, there were acquisition of nonrecurrent additional copy-number gains and losses in the high-grade dedifferentiated component not seen in the WDCS component (Fig. 3).

**FIGURE 4:**
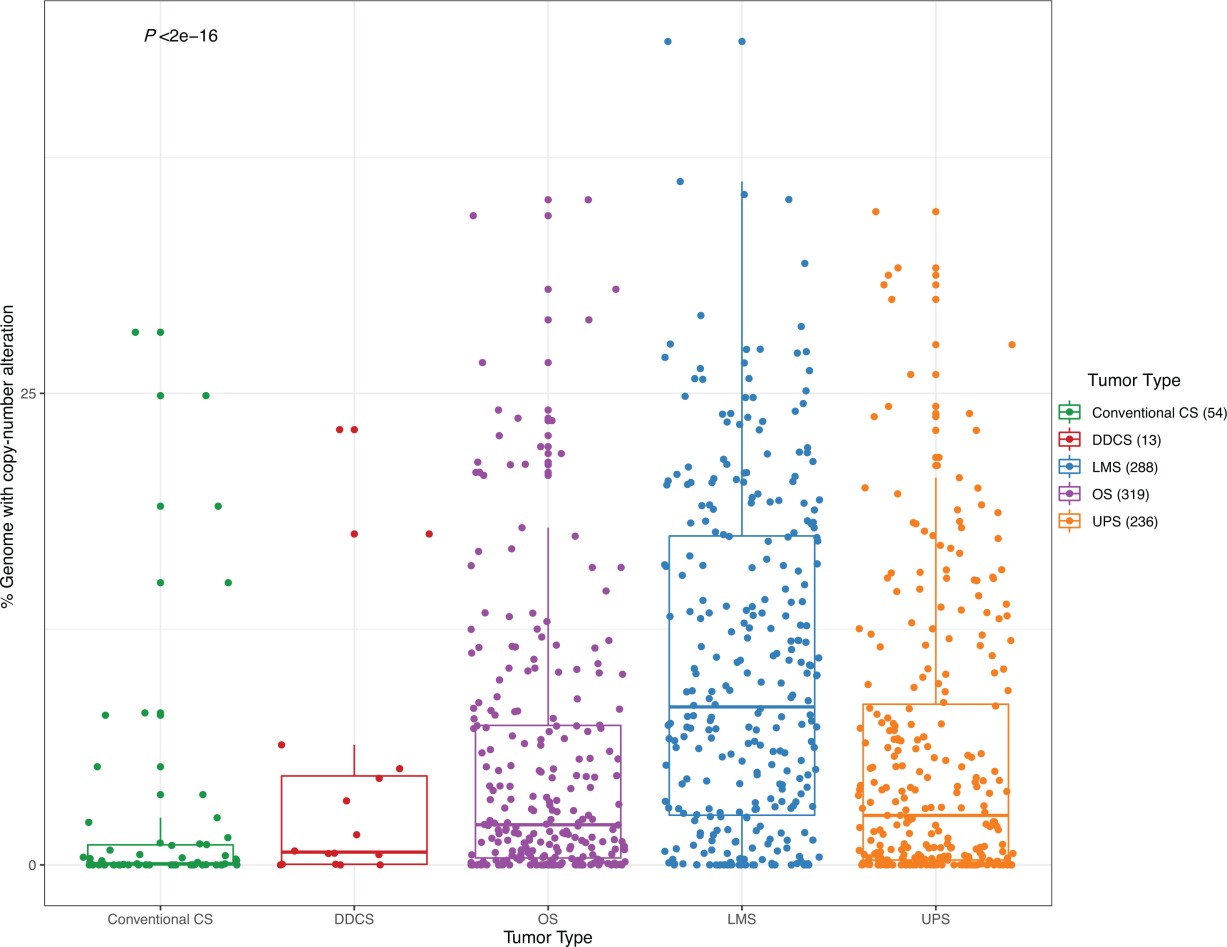
Percentage of genome CNAs in CS and high-grade sarcomas. Genome-wide CNA obtained by the MSK-IMPACT pipeline were quantified for conventional CS, DDCS, and compared with osteosarcomas (OS), LMS, and UPS, depicted by boxplots. Center line corresponds to the median; lower and upper hinges correspond to 25th and 75th percentiles; upper and lower whiskers correspond to 1.5 × interquartile range. Each dot represents individual cases. *P* value by ANOVA analysis.

Next, we analyzed and compared the percentages of genome-wide copy-number alterations among conventional CS and DDCS cases in comparison with other high-grade sarcomas. The percentage of genome involved by CNAs in DDCS was significantly lower than those in other high-grade sarcomas (osteosarcoma, LMS, UPS; Wilcoxon *P* < 0.0001; Fig. 4).

### Methylation Profiling and Differential Methylation in DDCS versus Conventional CS

**FIGURE 5:**
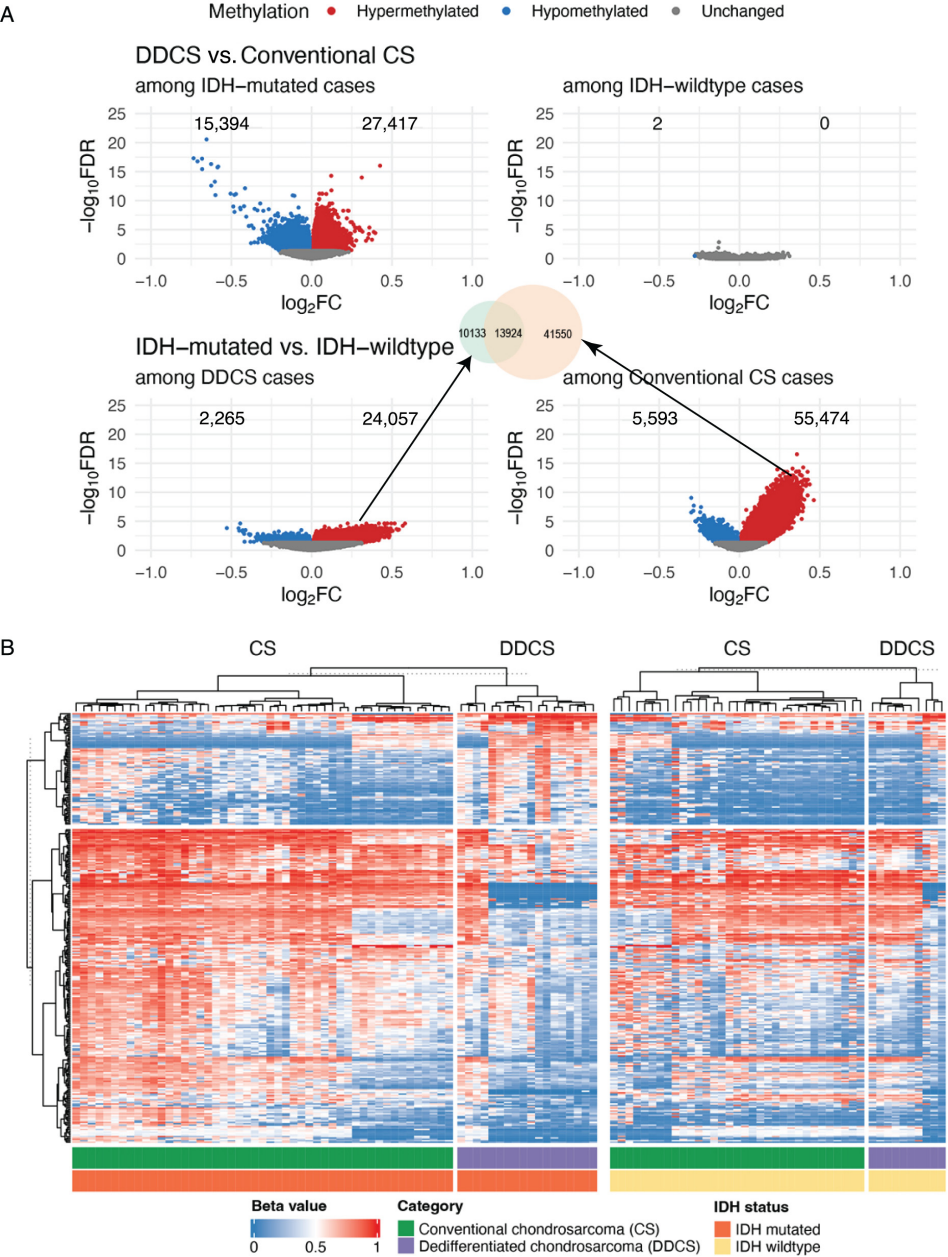
IDH-associated methylation in DDCS. Differential methylation analysis of DDCS versus conventional CS adjusted by IDH mutational status was performed. Methylation profiles of 94 conventional CS and 33 DDCS cases detected by the Illumina 450k or EPIC methylation array platforms, including cases from the MSKCC cohort, and external data from Nicolle 2019 and Koeslche 2019, were retrieved. Differential methylation analysis was performed on CpG sites between DDCS and conventional CS cases within IDH-mutated and IDH-wildtype groups. **A,** Volcano plots showing −log_10_ (FDR) against log_2_(fold change, FC) comparing methylation in DDCS versus conventional CS among IDH-mutated and IDH-wildtype cases, and IDH-mutated versus IDH-wildtype cases among DDCS and conventional CS. Differentially methylated sites are highlighted in red (hypermethylated) and blue (hypomethylated), respectively. FDR: false discovery rate (adjusted *P* value corrected by the Bonferroni–Holm method). FC: fold change. **B,** Heatmap represents beta values (ratio of the methylated probe intensity to the overall intensity—sum of methylated and unmethylated probe intensities) of the top 100,000 more variable CpG sites clustered by CS type and IDH1/2 mutational status.

Because CS is known to frequently harbor *IDH1/IDH2* mutations, which are known to cause global hypermethylation, we decided to examine and compare methylation profiles in conventional CS versus DDCS and how that is affected by *IDH1/IDH2* mutational status. In combination with external, publicly available methylation data of 82 conventional CS and 28 DDCS cases with known *IDH1/IDH2* status, we analyzed differentially methylated CpG sites (*P*_adjusted_ < 0.05) in DDCS versus conventional CS, adjusted by *IDH1/IDH2* mutational status. Comparing *IDH1/IDH2*-mutant versus -wildtype cases, we observed widespread *IDH1/IDH2* mutant–dependent hypermethylation of CpG sites among conventional CS cases. Importantly, the proportion of these *IDH1/IDH2*-associated hypermethylated sites were significantly reduced in the DDCS cases compared with conventional CS [24,057 (9.4%) vs. 55,474 (21.6%), *P* < 0.0001]. Furthermore, of the 55,474 *IDH1/IDH2*-associated hypermethylated sites in conventional CS, only 13,924 (25%) remained hypermethylated in DDCS (Fig. 5A and B), suggesting both a reduced and altered IDH-dependent methylation landscape in DDCS. In contrast, among IDH-wildtype cases, there were no significantly differentially methylated CpG sites comparing DDCS versus conventional CS cases (Fig. 5A and B). In other words, IDH-mutant DDCS exhibit methylation patterns that are distinct from IDH-mutant conventional CS.

**FIGURE 6:**
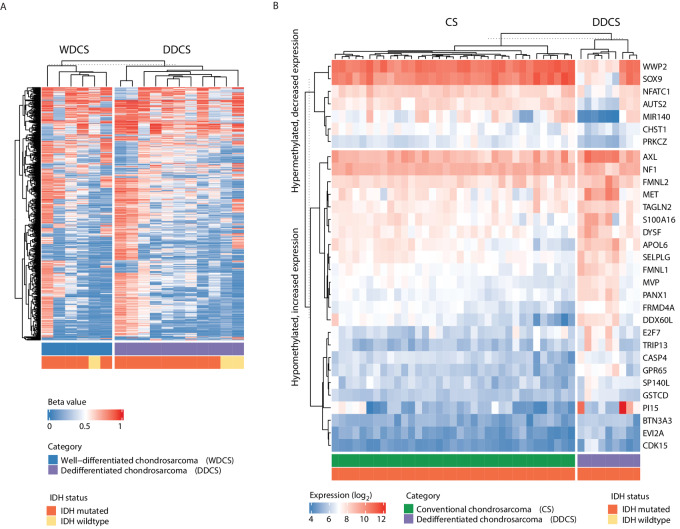
**A,** Methylation profiles of cartilaginous and sarcomatous components of DDCS. Microdissected WDCS components and high-grade noncartilaginous sarcomatous components of DDCS cases from the MSKCC cohort were subjected to methylation profiling by the Illumina 850k/EPIC array platform. Heatmap represents beta values of the CpG sites clustered by DDCS component subtype. **B,** Integrated differential methylation and expression analysis in DDCS. Genes corresponding to differentially methylated probes were matched to differentially expressed genes by intersecting hypermethylated genes to downregulated genes (decreased expression), and hypomethylated to upregulated genes (increased expression) in DDCS relative to conventional CS within the IDH-mutated group. Heatmap represents the top genes from this integrated analysis with a minimum log_2_ fold change of 1.5.

On the other hand, the macrodissected WDCS and high-grade sarcoma components in DDCS cases showed similar methylation profiles, regardless of *IDH1/IDH2* mutational status, suggesting that the observed *IDH1/IDH2*-associated methylation pattern is an early event in DDCS (Fig. 6A).

### Integrated Methylation and Expression Analysis

Next, to interrogate how such changes of methylation in DDCS affect gene expression, using external, publicly available Affymetrix gene expression data, we analyzed differentially methylated genes (*P*_adjusted_ < 0.05) in DDCS versus conventional CS, adjusted by *IDH1/IDH2* mutational status. Among 25,667 genes that were profiled, within the IDH-mutant group, there were 910 genes that were significantly upregulated and 1,602 that were significantly downregulated in expression in DDCS compared with conventional CS. In contrast, within the IDH-wildtype group, there were 243 genes that were significantly upregulated and 374 that were significantly downregulated in expression in DDCS compared with conventional CS.

We then matched these differentially expressed genes to the corresponding differentially methylated CpG sites by correlating the genes upregulated (increased expression) in DDCS to CpG sites hypomethylated in DDCS, and vice versa. Figure 6B depicts the expression of top genes that were hypomethylated and upregulated and those that were hypermethylated and downregulated in DDCS versus conventional CS.

**FIGURE 7:**
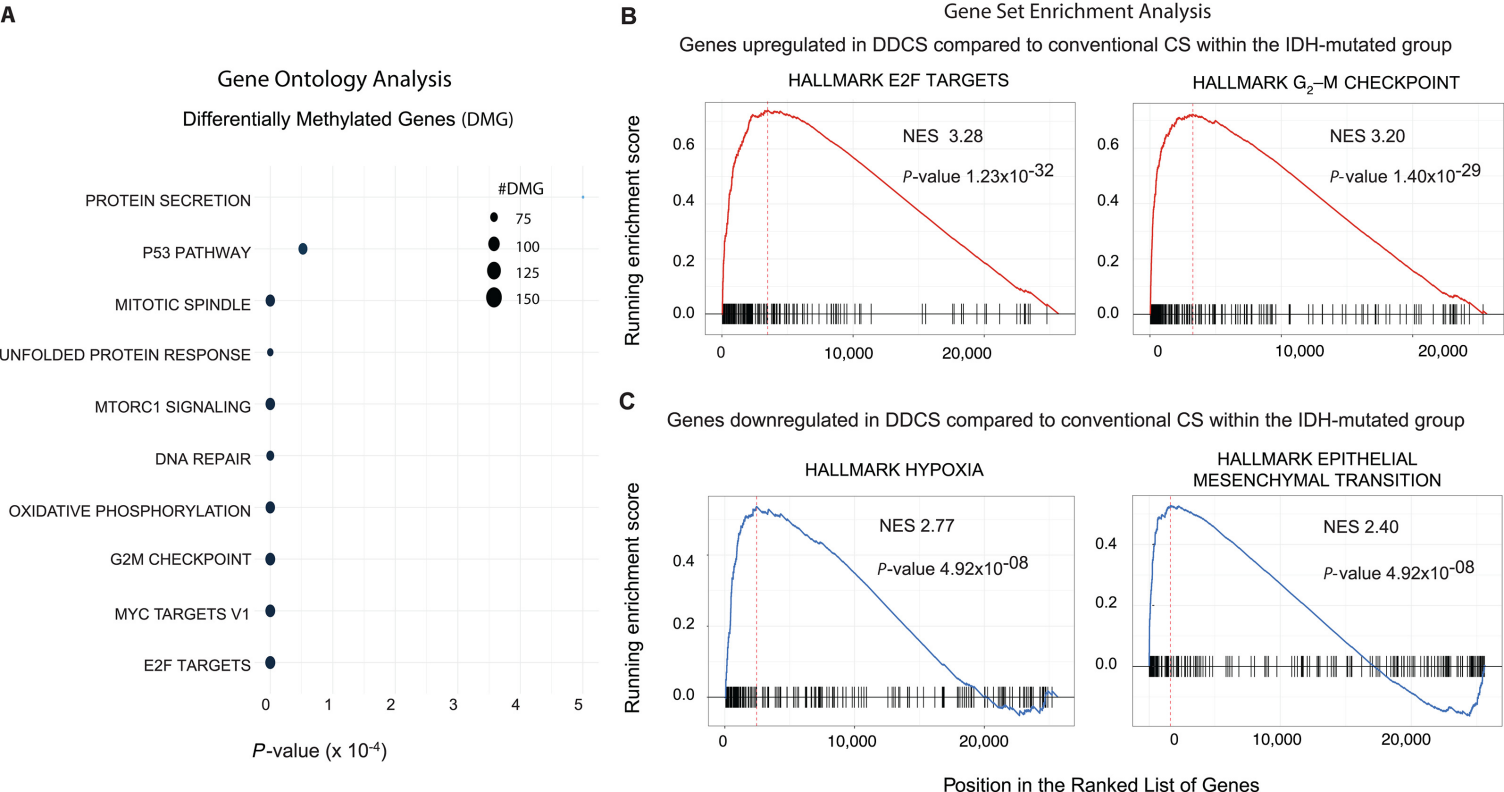
Pathway analysis of differentially methylated and expressed genes in DDCS compared with conventional CS. **A,** Gene ontology analysis of differentially methylated CpG sites (DMG) in DDCS versus conventional CS within the IDH-mutated grouop. Dot plot shows the pathways denoted by the significant gene sets. *P* values were adjusted by the Benjamini–Hochberg correction method. **B** and **C,** GSEA of differentially expressed genes in DDCS versus conventional CS within the IDH-mutated group. Shown are the top pathways for genes upregulated (**B**) and downregulated (**C**) in DDCS compared with conventional CS. *P* values were adjusted by the Benjamini–Hochberg correction method.

Gene ontology analysis showed that the differentially methylated sites between DDCS and conventional CS within the IDH-mutant group were enriched in genes associated with pathways involved with E2F targets, G_2_–M checkpoint, MYC targets, etc. (Fig. 7A). Concomitantly, GSEA of the genes upregulated in DDCS compared with conventional CS within the IDH-mutant group revealed enrichment of genes associated with pathways in G_2_–M checkpoint, E2F and MYC targets, and inflammatory/cytokine responses (Fig. 7B; Supplementary Table S1). Moreover, GSEA of the genes downregulated in DDCS compared with conventional CS within the IDH-mutant group revealed enrichment of genes associated with pathways involved in hypoxia, epithelial–mesenchymal transition, and metabolic responses (Fig. 7C; Supplementary Table S1).

## Discussion

We performed comprehensive, integrated genomic, and methylation profiling of a sizable cohort of DDCS cases, including macrodissected WDCS components, in comparison with conventional CS cases. By targeted DNA sequencing, *IDH1*/*IDH2* mutations were present in 36% conventional CS and 71% DDCS cases. Compared with conventional CS, DDCS had increased tumor mutation burden and higher frequencies of *TP53* and *TERT* promoter mutations and *CDKN2A/CDKN2B* copy-number losses.

In addition, paired analysis of macrodissected WDCS and the high-grade sarcoma components revealed *TERT* promoter mutations as common, early events in both components, but acquisition of additional copy-number gains and losses in the high-grade sarcoma component not seen in the WDCS component. These observations corroborate previous studies that showed frequent alterations in *CDKN2A* and *TP53* in DDCS (12), and that *IDH1/2* and *TERT* mutations are shared by both WDCS and high-grade sarcomatous components, while acquisition of *TP53* mutations and additional CNAs are seen mostly in the high-grade component only (14).

Despite phenotypic similarities, the percentage of genome involved by CNAs in DDCS was significantly lower than those in other high-grade sarcomas, that is, osteosarcoma, LMS, and UPS. To our surprise, DDCS does not seem to be more or as genomically complex compared with other high-grade sarcomas with complex karyotypes, such as osteosarcomas, despite sharing phenotypic features, and poor survival. This suggests that the biology and clinical behavior of DDCS may be driven by epigenetic factors rather than genotype/genomic complexity.

*IDH1/IDH2* mutations in cartilage tumors were associated with an aberrant epigenome, leading to global hypermethylation and downregulated expression of genes (8, 24, 31, 32). Expression of mutant IDH2 in mesenchymal progenitor cells led to DNA hypermethylation and an impairment in differentiation (33). This is due to increased production of the oncometabolite D-2-hydroxyglutarate (D-2-HG), an inhibitor of ten-eleven translocation (TET)-mediated DNA demethylation (32, 33). In human mesenchymal stem cells, D-2-HG has also been shown to promote chondrogenic over osteogenic differentiation (34). One study suggested that this dysregulation of differentiation by 2-HG–producing mutant IDH was by suppression of histone H3K9 demethylation, which repressed expression of lineage-specific differentiation genes (35). In human mesenchymal stem cells, induction of the *IDH1* R132C mutant led to enhanced expression of *SOX9* and *COL2A1* via increased H3K4me3 and inhibited expression of *ALPL* via increased H3K9me3 (36).

Nonetheless, the prognostic impact of *IDH1/IDH2* mutations is unclear (37). While some studies have suggested an association of *IDH1/IDH2* mutations with worse overall survival (38), others have not demonstrated such associations (24, 29). One study indicated that *IDH1/IDH2* mutations are associated with improved PFS but not OS in high-grade CS (39). Another recent study in central CS showed that although *TERT* mutations occur more frequently in *IDH2-*mutant compared with *IDH1-*mutant tumors, they are associated with worse survival in *IDH1-*mutant but not *IDH2-*mutant tumors (40).

Similar to conventional CS, about 50%–80% of DDCS also harbor *IDH1/IDH2* mutations in both WDCS and high-grade sarcomatous components (12, 16, 17), with no clear prognostic differences between IDH-mutant versus IDH-wildtype DDCS (17). The epigenetic alterations in DDCS, including methylation of CDKN2A isoforms and E-cadherin, shown in an early study could be accounted for by the increase in D-2-HG produced by mutant *IDH* in DDCS (41). Other studies demonstrated genetic alterations such as *SUZ12* or *EED* alterations in H3K27me3-deficient DDCS cases (42). Another study showed that L-2-HG, another enantiomer of 2-HG, is increased in hypoxia, leading to the increased methylation of histone repressive marks such as H3K9me3 (43, 44). Furthermore, a recent study showed increased expression of the HSPs *HSP70* and *HSP90* in DDCS (45), corroborating the findings of upregulation of HIF2α in CS progression (46).

We analyzed methylation profiling data from conventional CS and DDCS, combining our own cohort with external data. Then we performed differential methylation analysis between conventional CS versus DDCS, adjusted by *IDH1/IDH2* mutation status. Interestingly, we observed differentially methylated CpG sites between conventional versus DDCS only within the IDH-mutant cases but not within the IDH-wildtype cases. Furthermore, the WDCS and high-grade sarcoma components in DDCS showed similar methylation profiles. Concordant with previous studies, within the IDH-mutant group, we observed widespread hypermethylation of CpG sites among conventional CS cases. Importantly, differential methylation analysis revealed that *IDH1/IDH2* mutations were associated with a significantly reduced extent of hypermethylation in DDCS compared with conventional CS. Furthermore, the hypermethylated sites in DDCS were distinct from those in conventional CS. Then we performed GSEA of the genes associated with these differentially methylated CpG sites, and the topmost significant pathways were E2F targets, G_2_–M checkpoints, MYC targets, and inflammatory responses. We acknowledge the relatively small amplitude of the fold change and the discrepancy of sample sizes between DDCS and conventional CS. Nonetheless, there may a biological effect of this small absolute numerical fold change. Hundreds of thousands of CpG sites show variable methylation levels on a continuum. Deciphering the precise impact of how subtle amplitude of changes in methylation levels translate to alterations in gene expression would require detailed functional studies on specific genes.

It is well known that when WDCS transform to a DDCS, they lose the histologic appearance of cartilaginous differentiation and “convert to” other mesenchymal lineages, most frequently displaying morphologies akin to UPS or osteosarcoma; however, some cases can show leiomyosarcomatous, rhabdomyosarcomatous, or angiosarcomatous differentiation (1, 47, 48). Regardless of phenotype, the initial driver—*IDH1/IDH2* mutation—persists. We hypothesize that in *IDH1/2-mutant* tumors, the process of dedifferentiation is likely driven by a reversal of IDH-induced hypermethylation occurring at the early stage of WDCS, despite similar histologic appearance of a conventional CS. Such alterations of methylation pattern or *de novo* hypomethylation, we propose, is key to the progression to dedifferentiation, which ultimately leads to alteration of cellular lineage and therefore a dedifferentiated phenotype. Alternatively, rather than DDCS arising from conventional CS, our findings could instead support the theory of distinct cells-of-origin between DDCS and conventional CS, despite histologic similarities between WDCS of DDCS and conventional CS. It is possible that in a different cellular context in DDCS, mutant IDH-dependent inhibition of TET-mediated demethylation occurs at reduced number of and at alternative sites, resulting in epigenetic reprogramming in the progression to dedifferentiation. Further studies are needed to elucidate the mechanism and biological consequences of reduced and altered *IDH-*associated hypermethylation in DDCS. On the other hand, a recent study in central CS suggested that *IDH1-*mutant tumors displayed significantly higher degree of global hypermethylation compared with *IDH2-*mutant and IDH*-*wildtype tumors (40). Future studies looking into the differences in the methylation landscapes between *IDH1* versus *IDH2-*mutant DDCS may offer additional insights into the biology of different DDCS subtypes.

Importantly, such alterations in methylation patterns in DDCS also raise the question of the role of epigenetic therapies and IDH1/IDH2 inhibitors in the treatment of DDCS (49). This is especially relevant as preclinical studies have demonstrated activity of DNA methyltransferases and histone deacetylases inhibitors in the treatment of CS cell lines and xenografts (50). Furthermore, clinical trials looking at the use of mutant IDH inhibitors in advanced CS are currently ongoing. However, a recent phase I clinical study of ivosidenib, a selective mutant *IDH1* inhibitor, in patients with advanced *IDH1*-mutant CS showed PFS rates of 30% and 0% in DDCS versus 77% and 54% in conventional CS at 3 and 6 months, respectively. This suggests a different biology between DDCS and conventional CS despite sharing *IDH1* mutations (18), which is supported by the findings of the current study.

In conclusion, genomic profiling revealed enrichment of *TP53*, *TERT* promoter, and *CDKN2A/CDKN2B* alterations in DDCS. Integrated methylation and gene expression analysis revealed a reduction of *IDH1/2*-associated global hypermethylation and a distinct methylation and transcriptional landscape in DDCS, underpinning an important role in the pathogenesis of dedifferentiation in CS.

## Supplementary Material

Supplementary Figure FS1 Overall and progression-free survival in DDCS patients with or without TERT alterations

Supplementary Table TS1 Gene set enrichment analysis
