# Supplementary material for: Distinct IDH1/2-associated Methylation Profile and Enrichment of TP53 and TERT Mutations Distinguish Dedifferentiated Chondrosarcoma from Conventional Chondrosarcoma
Source: Cancer Res Commun. 2023 Mar 14;3(3):431–43. doi: 10.1158/2767-9764.CRC-22-0397 (PMC10013202; doi:10.1158/2767-9764.CRC-22-0397)
Supplement: Supplementary Figure FS1 — Overall and progression-free survival in DDCS patients with or without TERT alterations [file crc-22-0397-s01.pdf]

A

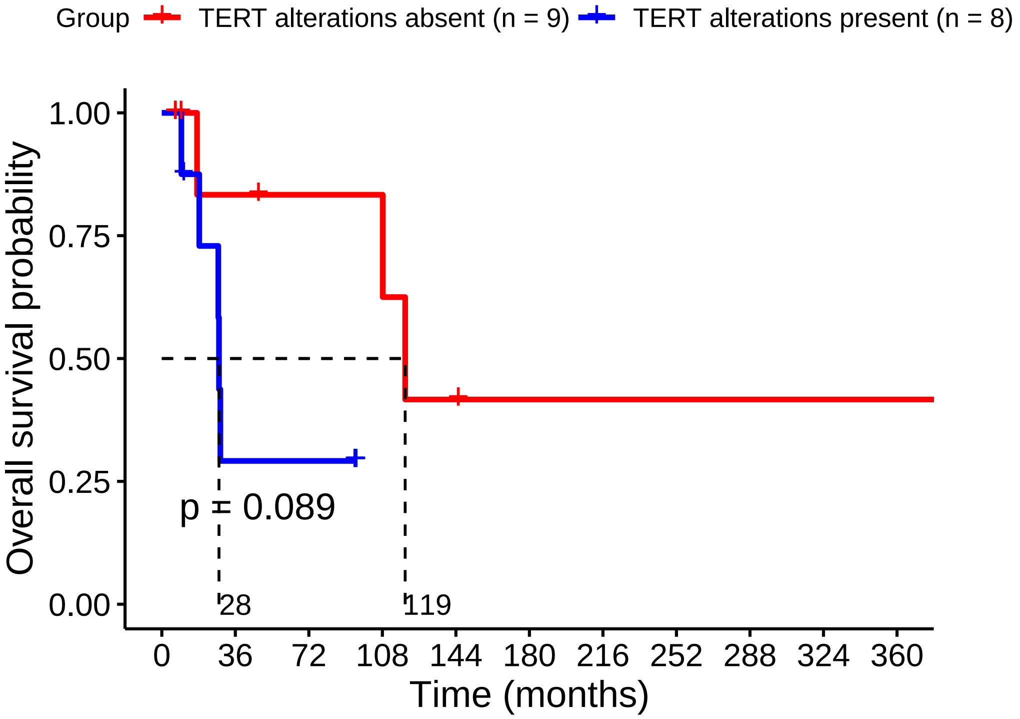

B

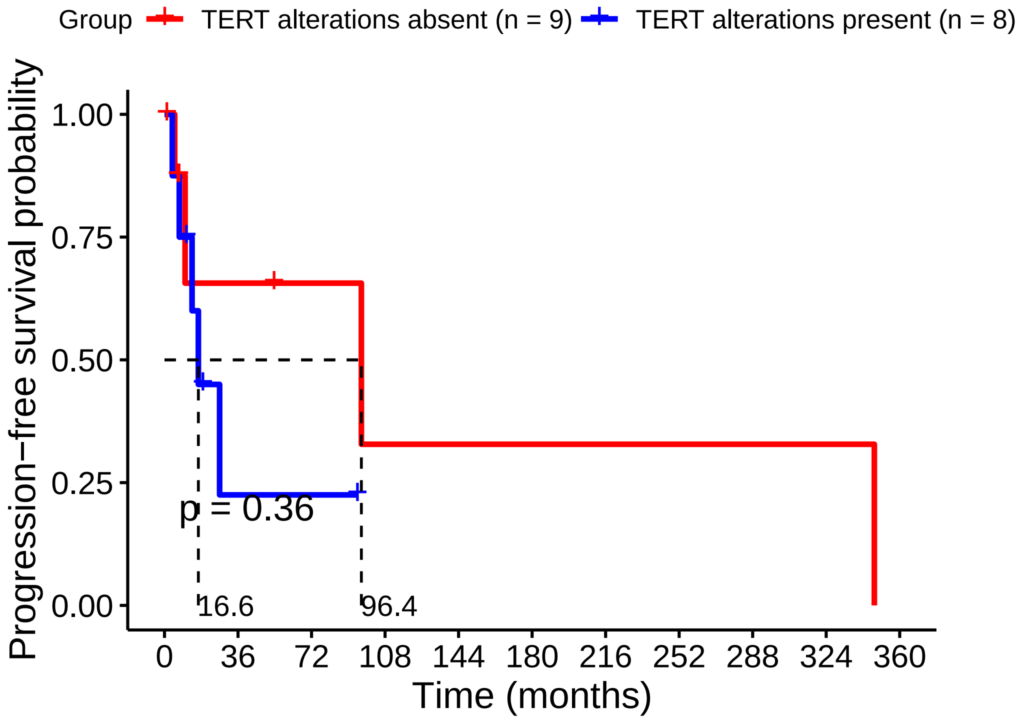

Supplementary Figure S1.
Kaplan-Meier curves depicting overall (A) and progression-free (B) survival of dedifferentiated chondrosarcoma patients with TERT promoter mutations versus those without TERT promoter mutations (log-rank P test). Dotted lines indicate median survival.
